# Knockdown of Circ_SLC39A8 protects against the progression of osteoarthritis by regulating miR-591/IRAK3 axis

**DOI:** 10.1186/s13018-021-02323-7

**Published:** 2021-03-03

**Authors:** Jizhe Yu, Yushuang Qin, Naxin Zhou

**Affiliations:** 1 Department of Orthopaedics, Yichang Central People’s Hospital, 183 Yiling Avenue, Wujiagang District, Yichang City, 443003 Hubei Province P. R. China; 2 Department of Nuclear Medicine, Yichang Central People’s Hospital, 183 Yiling Avenue, Wujiagang District, Yichang City, 443003 Hubei Province P. R. China

**Keywords:** Osteoarthritis, Circ_SLC39A8, miR-591, IRAK3

## Abstract

**Background:**

The dysregulation of circular RNAs (circRNAs) has been identified in various human diseases, including osteoarthritis (OA). The purpose of this study was to identify the role and mechanism of circ_SLC39A8 in regulating the progression of OA.

**Methods:**

The expression levels of circ_SLC39A8, miR-591, and its potential target gene, interleukin-1-receptor-associated kinase 3 (IRAK3), were identified by quantitative real-time polymerase chain reaction (qRT-PCR). Cell viability and apoptosis were determined by Cell Counting Kit-8 (CCK-8) assay and flow cytometry, respectively. The relationship between miR-591 and circ_SLC39A8 or IRAK3 was predicted by bioinformatics tools and verified by dual-luciferase reporter.

**Results:**

Circ_SLC39A8 and IRAK3 were upregulated and miR-591 was downregulated in OA cartilage tissues. Knockdown of circ_SLC39A8 inhibited apoptosis and inflammation in OA chondrocytes, while these effects were reversed by downregulating miR-591. Promotion cell viability effects of miR-591 were partially reversed by IRAK3 overexpression.

**Conclusion:**

Our findings indicated that knockdown of circ_SLC39A8 delayed the progression of OA via modulating the miR-591-IRAK3 axis, providing new insight into the molecular mechanisms of OA pathogenesis.

## Introduction

Osteoarthritis (OA) is the most common joint disease and is recognized as one of the leading causes of pain and disability among the elderly [1, 2]. OA is primarily characterized by degradation of the articular cartilage, as well as subchondral bone sclerosis, and osteophyte formation. OA is a global health problem and the most common joint disorder disease [3, 4]. The Osteoarthritis Research Society International (OARSI) pointed out that OA affects 240 million people worldwide [5]. Among people over 60, about 10% of men and 18% of women have OA. Studies have pointed out that 19% of people over 45 years of age have X-rays showing OA [6]. The risk factors of OA are still increasing, including age, obesity, gender, genetics, low-grade systemic inflammation, and increased joint biomechanical load [7].

The manifestations of OA cartilage are mainly accumulation of senescent cells, cell loss, and extracellular matrix destruction. Inflammation response is involved in many pathophysiological conditions related to aging, including OA [8]. Elderly patients with OA have higher levels of inflammatory biomarkers. Inflammatory cytokines interfere with the catabolism of bones and joints, causing severe pain and even disability [9].

Circular RNAs (circRNAs) are a special type of non-coding RNA that was first identified 20 years ago and have attracted significant attention in recent years due to their diverse activities [10]. circRNA is characterized by the covalently closed loop structure without 5′-end cup and 3′-end ploy-A tail [11]. circRNA can modulate gene expression, and they are closely involved in multiple diseases, including OA [12]. For example, Circ_DHRS3 [13], CircADAMTS6 [14], and circANKRD36 [15] played pivotal roles in regulating OA chondrocyte growth, differentiation, and apoptosis. hsa_circ_0002782 (chr4:103225473-103236987) is derived from back-splicing of SLC39A8 transcript and was suggested to be upregulated in OA. However, the exact roles and regulatory mechanism of circ_SLC39A8 in OA have not been reported.

Generally, circRNAs act as competitive endogenous RNAs (ceRNAs) or microRNA (miRNA) sponges, competing for miRNA binding and affecting miRNA function [16]. The miRNAs usually negatively regulate gene expressions by binding specific mRNAs in their 3′-untranslated regions (UTRs) based on sequence complementation to promote the degradation of target mRNAs or to inhibit their translation. Dysregulation of miRNAs is involved in multiple pathological processes of various cardiovascular diseases, including OA [17–19]. miR-591 has been shown to be involved in the regulation of multiple diseases, including breast cancer progression [20], hepatocellular carcinoma [21], and human heart failure [22]. For example, it has been reported that miR-591 inhibited cell malignancy and glycolysis by targeting HK2 in breast cancer [23]. However, the role of miR-591 in the progression of OA was not fully understood. IRAK3 belongs to the IL receptor-associated kinase (IRAK) family involved in inhibiting Toll-like receptor signaling [24]. The role of IRAK3 in the progression of OA was not reported in previous studies and thus needs further research.

In this research, circ_SLC39A8, miR-591, and IRAK3 abundance were measured in OA cartilage tissues. Moreover, we explored the effects of circ_SLC39A8, miR-591, and IRAK3 on cell viability, apoptosis, inflammation, and ECM degradation and determined their relationships in OA chondrocytes. Collectively, our research focused on uncovering the role of the circ_SLC39A8/miR-591/IRAK3 axis in OA chondrocytes.

## Materials and methods

### Specimens collection

Human cartilage tissues were collected from OA patients (*n*=30, age 61.04 ±4.809 years; 18 females, 7 males) who underwent total knee replacement surgery and from traumatic amputees (*n*=20, age 61.04 ±4.809 years; 18 females, 7 males) without rheumatoid arthritis or OA. All subjects understood and signed the informed consent form before participation. All the tissue samples were instantly frozen in liquid nitrogen after surgical resection and were preserved at – 80 °C until further use.

### OA chondrocyte isolation and culture

Chondrocytes were isolated from OA cartilage tissues, as previously described [25]. Briefly, cartilage tissues were cut into approximate 1-mm^3^ segments and digested enzymatically using 0.25% trypsin (Gibco, Life Technologies, Paisley, UK) for 0.5 h followed by 0.2% type II collagenase (Invitrogen, Carlsbad, CA, USA) for 3 h at 37 °C. Cells were maintained in Dulbecco’s modified Eagle medium (DMEM; Gibco, Carlsbad, CA, USA) containing 10% fetal bovine serum (FBS; Gibco), 100 U/ml penicillin, and 100 mug/ml streptomycin in a 5% CO_2_ incubator at 37 °C. Chondrocytes were treated with IL-1β (10 ng/ml) for 24 h to establish the OA model in vitro [26].

### Transfection

The small interfering RNAs (siRNAs) against circ_SLC39A8 (si-circ_SLC39A8#1, si- circ_SLC39A8#2, and circ_SLC39A8#3) and corresponding control (si-NC), miR-591 mimic (miR-591) and corresponding control (NC), miR-591 inhibitor (anti-miR-591) and corresponding control (anti-NC), and IRAK overexpression vector (IRAK) and corresponding control (vector) were purchased from Genepharma (Shanghai, China), and then, these oligonucleotides or vectors were transfected into chondrocytes using Lipofectamine 3000 (Invitrogen) for 48 h before propofol treatment.

### RNase R treatment

To determine the stabilization of circ_SLC39A8, 10 mug RNA extracted from chondrocytes was incubated with RNase R (4 U/mug; Epicentre Biotechnologies, Madison, WI, USA) or not at 37 °C for 1 h. Later, the relative expression of circ_SLC39A8 and LC39A8 mRNA was examined by qRT-PCR.

### Quantitative real-time polymerase chain reaction (qRT-PCR)

Total RNA was isolated from using the TRIzol Reagent (Invitrogen). RNA (1 mug) was reversely transcribed into cDNA using the PrimeScript RT reagent kit (TakaraBio, Tokyo, Japan) and a TaqMan miRNA reverse transcription kit (Applied Biosystems, Foster City, CA, USA). qRT-PCR was conducted with SYBR-Green Real-Time PCR Kit (Takara, Otsu, Japan). The amplification conditions were 1 cycle of 94 °C for 5 min followed by 35 cycles of 94 °C for 30 s, 59 °C for 35 s, and 72 °C for 45 s. The primers used for PCR amplification and nucleotide sequencing are listed as follows: circ_SLC39A8, 5′-GAACCAGTCACACCTGCATC-3′ (F) and 5′-TGGCTGCACATCACTCTGTA-3′ (R); miR-591, 5′-GAGGTAGACCATGGGTTCTCA-3′ (F) and 5′-AGCCAGGGAGTCCACAGTTA-3′ (R); IRAK3, 5′-TTGGTCCTGGGCACAGAAA-3′ (F) and 5′-AATAGCTCGACGATGTCCCAT-3′ (R); GAPDH, 5′-AGAAAAACCTGCCAAATATGATGAC-3′ (F) and 5′-TGGGTGTCGCTGTTGAAGTC-3′ (R); and U6, 5′-CTCGCTTCGGCAGCACATATACT-3′ (F) and 5′-ACGCTTCACGAATTTGCGTGTC-3′ (R). Relative gene expression was calculated by the 2^−ΔΔCt^ method. Internal references of mRNA and miRNA were GAPDH and U6, respectively.

### Cell viability assay

Cell counting Kit-8 (CCK-8; Beyotime, Jiangsu, China) was employed for measuring cell viability. In short, OA chondrocytes (4×10^4^ cells/well) were placed in 96-well plates. CCK-8 (10 μL) was added to each well at pointed times. After incubation for 2–3 h, the absorbance at 450-nm wavelength was utilized to assess cell viability.

### Flow cytometry

Cell apoptosis was detected with an Annexin V-fluorescein isothiocyanate (FITC)/propidium iodide (PI) Apoptosis Detection kit (KeyGEN Biotech, Jiangsu, China) according to the manufacturer’s protocol. Chondrocytes in different groups were washed in cold PBS, re-suspended in 1x annexin-binding buffer, and stained with Annexin V-FITC and PI. Chondrocytes were counted by flow cytometer (Partec AG, Arlesheim, Switzerland) after incubation in dark for 15 min and subjected to calculate the percentage of apoptotic cells.

### Western blot assay

Proteins in chondrocytes were extracted using radioimmunoprecipitation assay (RIPA) lysis buffer (Beyotime, Haimen, China) containing phenylmethylsulfonyl fluoride (PMSF). And the protein concentration was quantified by BCA Protein Quantification Kit (Solarbio, Beijing, China). After separated by sodium dodecyl sulphate-polyacrylamide gel electrophoresis (SDS-PAGE), proteins were transferred onto polyvinylidene fluoride (PVDF) membranes (Millipore, USA). Next, the membranes were blocked with 5% non-fat milk at room temperature for 2 h and incubated overnight at 4 °C with primary antibodies: Bcl-2 (Abcam, 1:500), Bax (Abcam, 1:500), caspase-3 (Abcam, 1:500), cleaved caspase-3 (Abcam, 1:500), caspase-9 (Abcam, 1:500), cleaved caspase-9 (Abcam, 1:500), GAPDH (Abcam, 1:500), TNF-α (Abcam, 1:500), IL-1β (Abcam, 1:500), IL-6 (Abcam, 1:500), MMP-3 (Abcam, 1:500), COL2A1 (Abcam, 1:500), and IRAK3 (Abcam, 1:500). GAPDH was the internal control. After Tris-buffered saline Tween-20 (TBST) washing three times, the membranes were further cultivated with secondary antibodies for 2 h at room temperature. Finally, the immunoreactive bands were visualized using an enhanced chemiluminescence (ECL) detection system (Thermo Fisher Scientific, NY).

### Dual-luciferase reporter assay

The potential complementary sequence of miR-591 and circ_SLC39A8 or IRAK3 was predicted by the starBase database (http://starbase.sysu.edu.cn/index.php). Partial sequences of circ_SLC39A8 or IRAK3 3′UTR containing wild-type (wt) or mutant (mut) miR-591 binding sites were synthesized and then cloned into the pmirGLO Dual-luciferase vectors (GenePharma, Shanghai, China), namely circ_SLC39A8-wt, circ_SLC39A8-mut, IRAK3-wt, and IRAK3-mut. OA chondrocytes were co-transfected with the constructed luciferase vector (wt or mut) and NC or miR-591 for 48 h. Luciferase activity was measured via the Dual-Luciferase Reporter Assay System (Promega, Madison, WI, USA) and normalized to the activity of Renilla luciferase.

### Bioinformatic analysis

The prognostic roles of GART in GC were analyzed using the GEPIA database. GART gene was submitted to the GEPIA database and the expression value of GART between GC and normal tissue was further analyzed. Then, the relations of overall survival (OS) rates with the expression of GART in GC were computed by using the GEPIA database.

### Statistical analysis

Each experiment was repeated three times. All statistical analyses were performed using SPSS version 22 (SPSS, Chicago, IL, USA). Student’s *t* test and one-way analysis of variance (ANOVA) and Bonferroni-Dunn post hoc tests were applied to analyze the statistical differences. Statistical significance was considered when *P*<0.05.

## Results

### Circ_SLC39A8 was upregulated in OA cartilage tissues

**Fig. 1:**
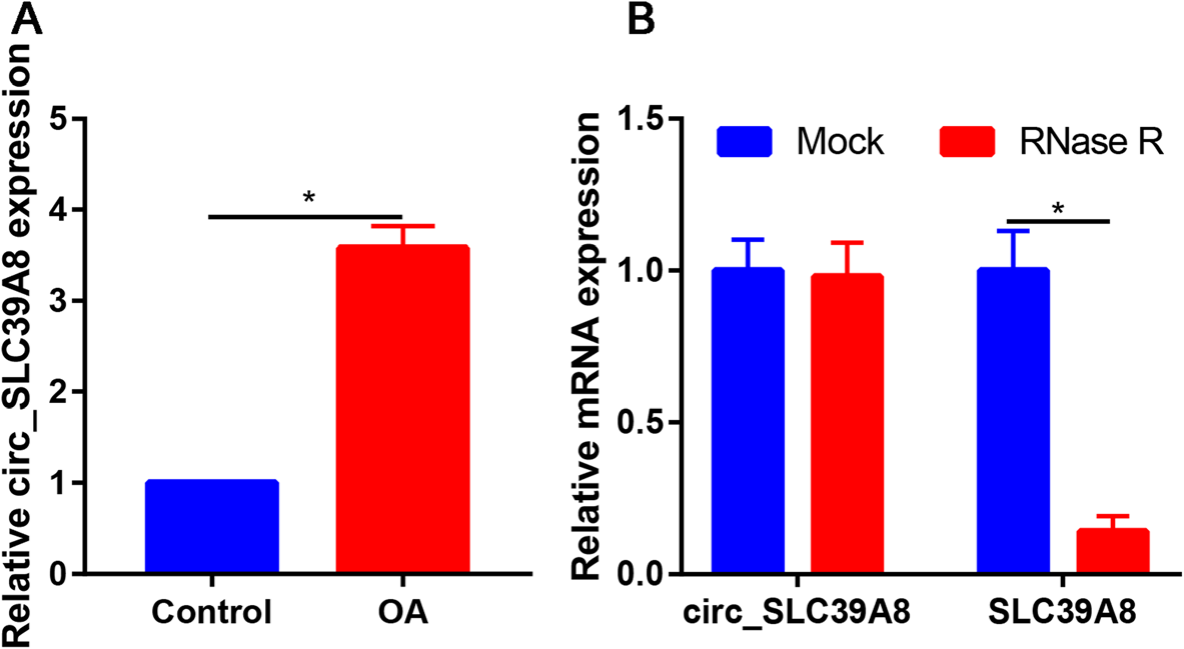
Circ_SLC39A8 expression was increased in OA cartilage tissues. **a** The expression of circ_SLC39A8 was determined by qRT-PCR analysis in OA cartilage tissues and normal cartilage tissues. **b** The levels of circ_SLC39A8 and linear mRNA were determined after treatment of RNase R by qRT-PCR in OA chondrocytes. *P<0.05

As illustrated in Fig. 1a, compared with normal cartilage, the circ_SLC39A8 expression was significantly increased in OA cartilage tissues with statistically significant (Fig. 1a). As displayed in Fig. 1b, RNase R treatment did not affect circ_SLC39A8 levels but affect SLC39A8 level, because RNase R is an exoribonuclease that can degrade RNA from its 3–5′ end but does not act on circRNAs.

### Knockdown of circ_SLC39A8 increased cell viability and inhibited apoptosis, inflammation, and ECM degradation in OA chondrocytes

**Fig. 2:**
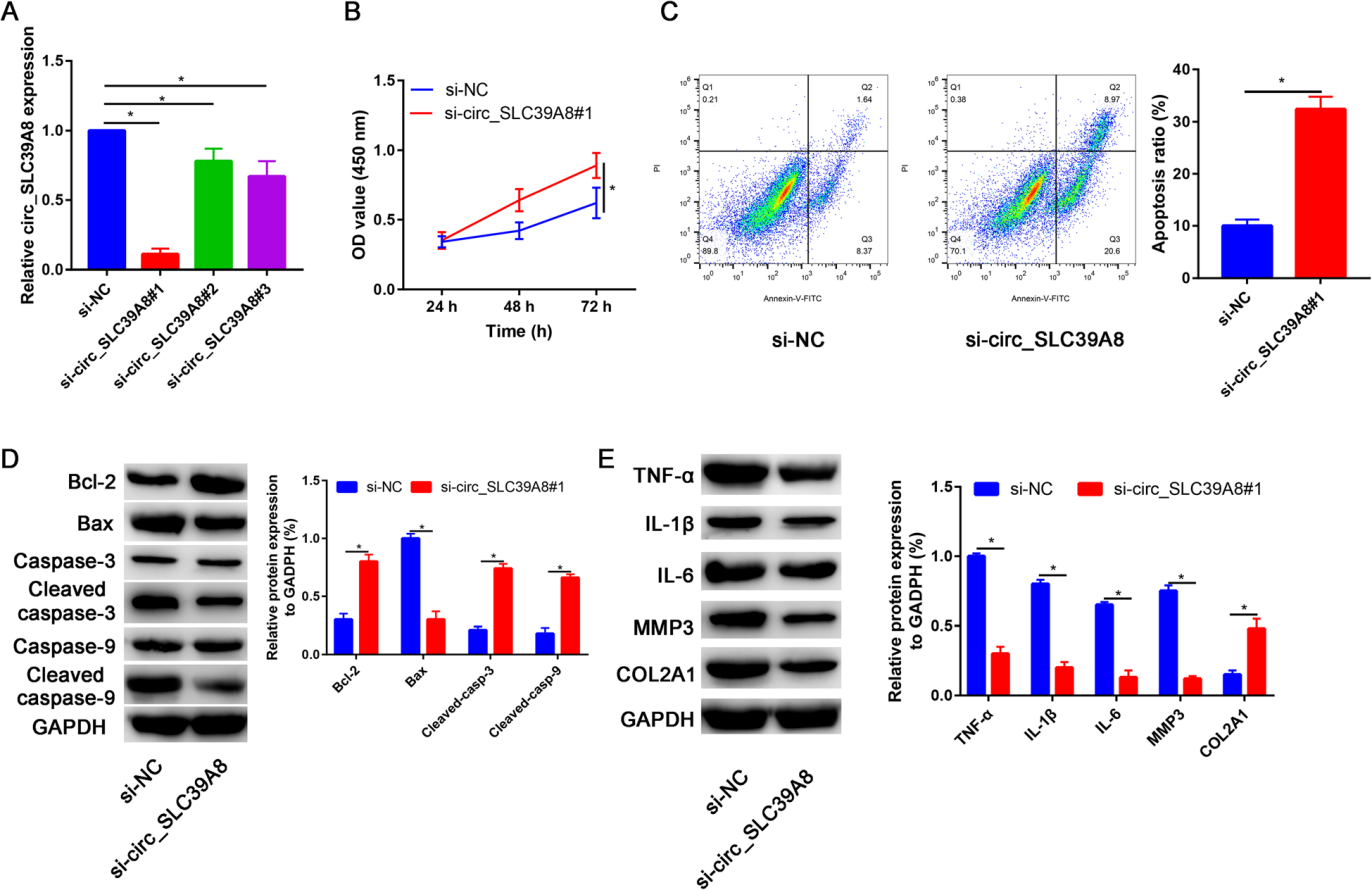
Circ_SLC39A8 silence increased cell viability and suppressed apoptosis, inflammation, and ECM degradation in OA chondrocytes. **a** Knockdown efficiency of circ_SLC39A8 was determined by qRT-PCR in OA chondrocytes transfected with si-NC, si- circ_SLC39A8#1, si- circ_SLC39A8#2, and si- circ_SLC39A8#3. **b**–**e** OA chondrocytes were transfected with si-NC or circ_SLC39A8#1. **b** Cell viability was assessed by CCK-8 assay. **c** Cell apoptosis was examined using flow cytometry analysis. **d** Western blot assay was conducted to measure the protein levels of Bcl-2, Bax, caspase 3, C-caspase 3, caspase 9, and C-caspase 9. **e** The protein levels of TNF-α, IL-1β, IL-6, MMP3, and collagen type II were detected by western blot analysis. *P<0.05

To explore the effect of circ_SLC39A8 on OA progression, knockdown of circ_SLC39A8 was constructed and the knockdown efficiency was determined by qRT-PCR. Si-circ_SLC39A8#1 displayed the highest silencing efficiency and was therefore used to transfect chondrocytes for further analysis (Fig. 2a).

CCK-8 assay revealed that si-circ_SLC39A8 enhanced cell viability in OA chondrocytes at 48 h and 72 h (Fig. 2b).

Moreover, si-circ_SLC39A8 significantly increase the apoptosis rate than si-NC (Fig. 2c).

Besides, the relative expression levels of apoptosis-related proteins, including Bcl-2, Bax, cleaved-caspase 3, and cleaved-caspase 9, were analyzed by western blot assay.

As presented in Fig. 2d, si-circ_SLC39A8 increased the protein level of Bcl-2 and decreased the protein expression of Bax, cleaved caspase 3/caspase 3 ratio, and cleaved caspase-9/caspase-9 ratio. Besides, pro-inflammatory cytokines (TNF-α, IL-1β, and IL-6), COL2A1, and MMP3 were detected by western blot assay.

The results showed that si-circ_SLC39A8 decreased the protein levels of TNF-α, IL-1β, IL-6, and MMP3 while increasing the protein expression of collagen type II (Fig. 2e). These results indicated that circ_SLC39A8 played a significant role in regulating cell viability, apoptosis, inflammatory response, and ECM degradation of OA chondrocytes.

### circ_SLC39A8 acted as a sponge of miR-591

**Fig. 3:**
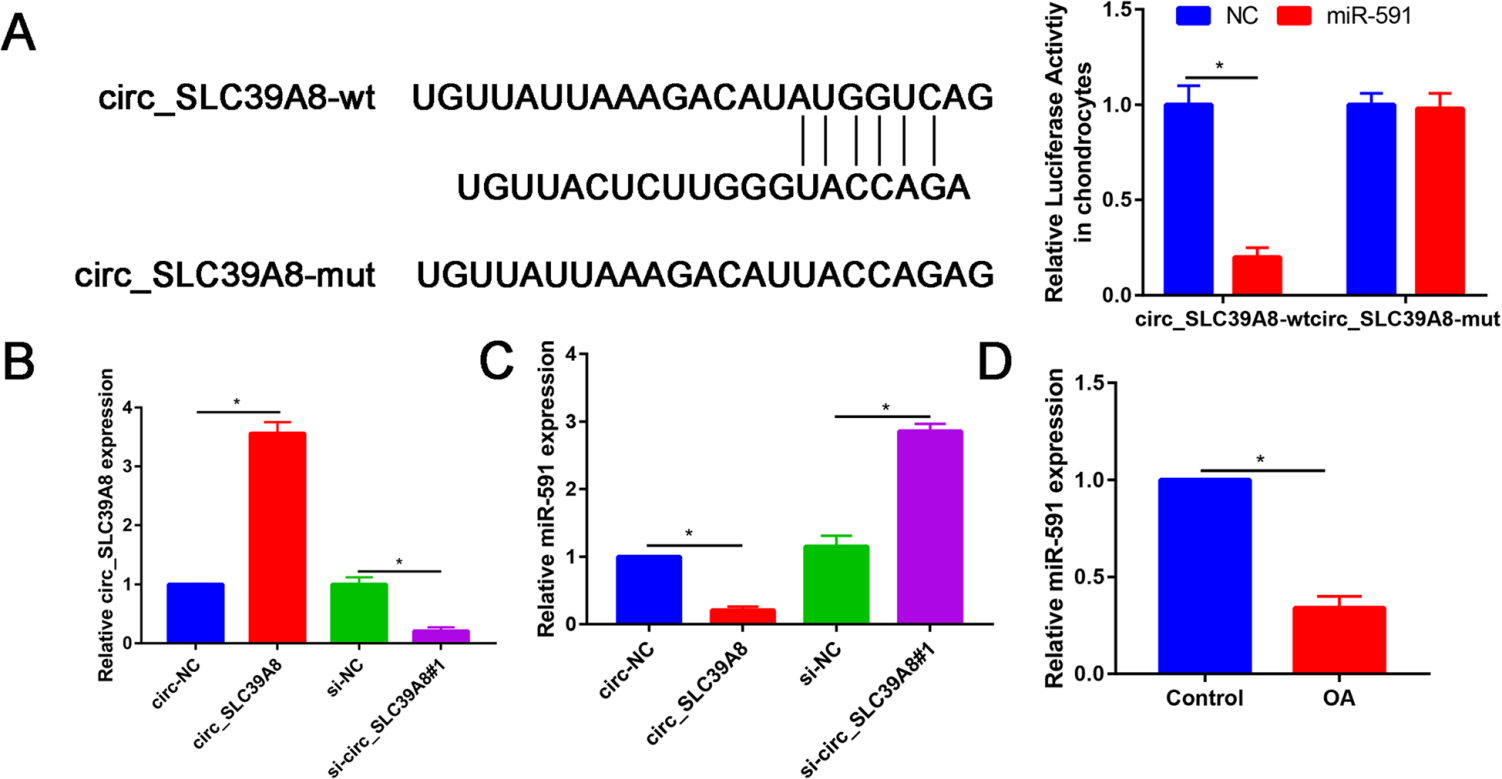
Circ_SLC39A8 directly interacted with miR-591. **a** The putative binding sites between circ_SLC39A8 and miR-591 and dual-luciferase luciferase reporter assay were used to detect the luciferase activity in OA chondrocytes co-transfected with circ_SLC39A8-wt or circ_SLC39A8-mut and NC or miR-591. **b** The expression levels of circ_SLC39A8 in OA chondrocytes transfected with circ-NC, circ_SLC39A8, si-NC, or si-circ_SLC39A8#1. **c** The expression levels of miR-591 in OA chondrocytes transfected with circ-NC, circ_SLC39A8, si-NC, or si-circ_SLC39A8#1. **d** The expression of miR-591 was analyzed in OA cartilage tissues and normal cartilage tissues by qRT-PCR. *P<0.05

To determine whether circ_SLC39A8 could serve as a sponge for miRNA, the potential targets of circ_SLC39A8 were predicted by starBase. First, the results of bioinformatics analysis starBase (http://starbase.sysu.edu.cn/index.php) suggested that miR-330-5p, which contained the putative binding sites, was the target of circ_SLC39A8 (Fig. 3a). Dual-luciferase reporter was conducted to confirm this prediction. The results show that overexpression of miR-591 significantly reduced the relative luciferase activity of the circ_SLC39A8 3′UTR wt plasmid, but did not change the luciferase activity of the circ_SLC39A8 3′UTR mut plasmid (Fig. 3a, *P* < 0.05).

qRT-PCR results indicated that si-circ-SLC39A8 reduced the expression of circ-SLC39A8, and transfection of circ-SLC39A8 increased the expression of circ-SLC39A8, suggesting that si-circ-SLC39A8 and circ-SLC39A8 were successfully transfected into chondrocytes (Fig. 3b).

Compared with circ-NC, circ-SLC39A8 significantly decreased the miR-591 expression, while the contrary trend was found for si-circ-SLC39A8 (Fig. 3c).

Then, we compared the expression level of miR-591 in OA cartilage and normal cartilage. The result found that miR-591 was significantly decreased in OA cartilage than normal cartilage (*P*<0.05, Fig. 3d).

### miR-591 knockdown reversed the effects of si-circ_SLC39A8#1 on cell viability, apoptosis, inflammation, and ECM degradation in OA chondrocytes

**Fig. 4:**
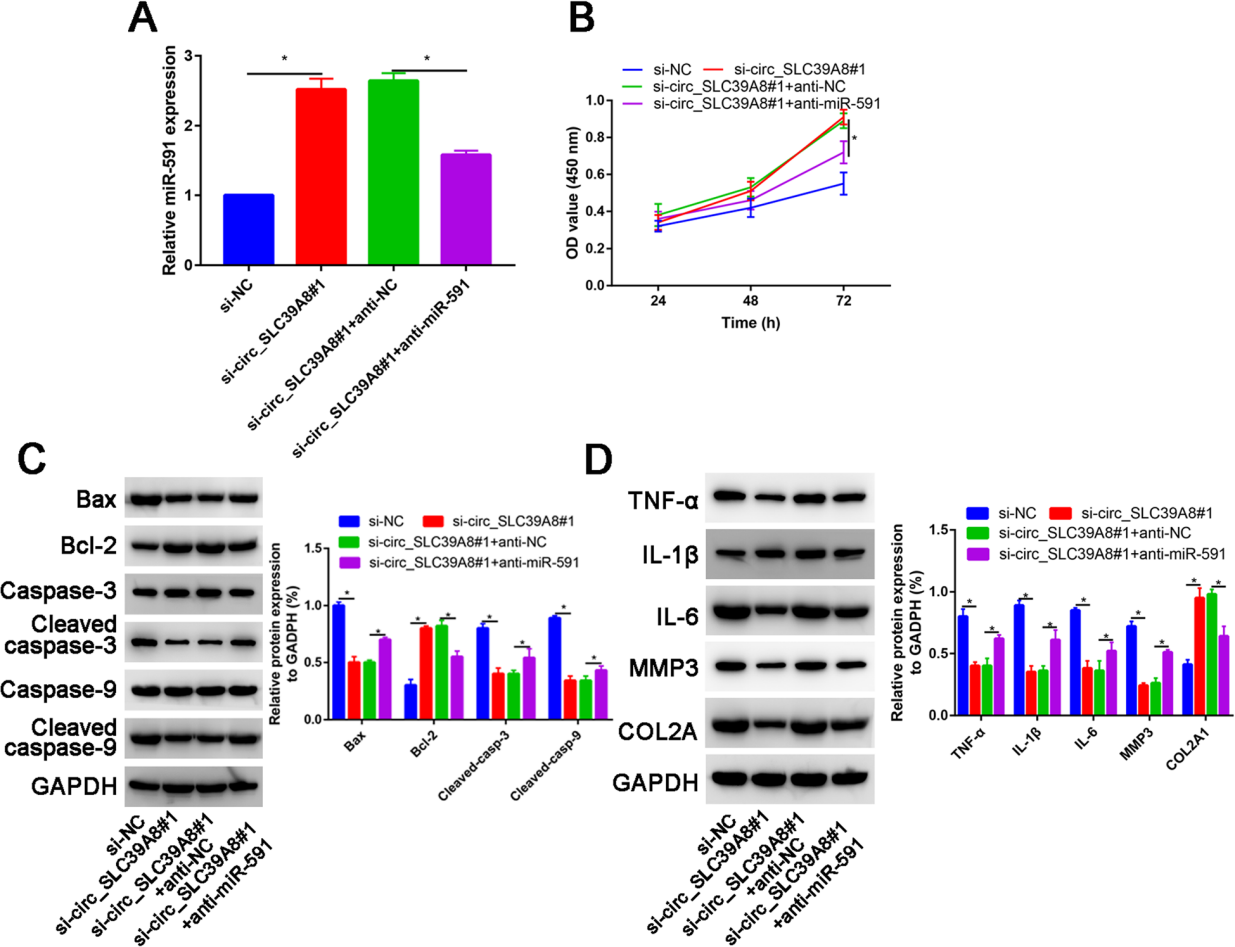
Circ_SLC39A8 exerted its functions by sponging miR-591 in OA chondrocytes. OA chondrocytes were transfected with si-NC, si-circ_SLC39A8#1, si-circ_SLC39A8#1 + anti-NC, or si-circ_SLC39A8#1 + anti-miR-591. **a** The level of miR-591 was examined by qRT-PCR. **b** CCK-8 assay was conducted to evaluate cell viability. **c** Protein levels of Bcl-2, Bax, caspase 3, cleaved caspase 3, caspase 9, and cleaved caspase 9 were examined by western blot assay. **d** The protein levels of TNF-α, IL-1β, IL-6, MMP3, and collagen type II were determined using western blot analysis. *P<0.05

Compared with si-NC, si-circ_SLC39A8#1 significantly increase miR-591 expression, while co-cultured si-circ_SLC39A8#1 and anti-miR-591 partially downregulated miR-591 expression (Fig. 4a).

CCK-8 assay indicated that the promoting effect of circ_SLC39A8 downregulation on cell viability was partially abolished by anti-miR-591 (Fig. 4b).

Anti-miR-591 abated the effect of circ_SLC39A8 knockdown on promoting Bcl-2 expression and reducing Bax expression, C-caspase 3/caspase 3 ratio, and C-caspase 9/caspase 9 ratio (Fig. 4c). In addition, the reduction of TNF-α, IL-1β, IL-6, and MMP3 expression and promotion of COL2A1 expression caused by transfection with si- circ_SLC39A8#1 were reversed by co-transfection with anti-miR-591 (Fig. 4d).

### IRAK3 was a direct target gene of miR-591

The putative binding sites for miR-591 within the 3′UTR of IRAK3 were identified using the Targetscan algorithm (targetscan.org). Moreover, dual-luciferase reporter assay was performed to validate target prediction.

**Fig. 5:**
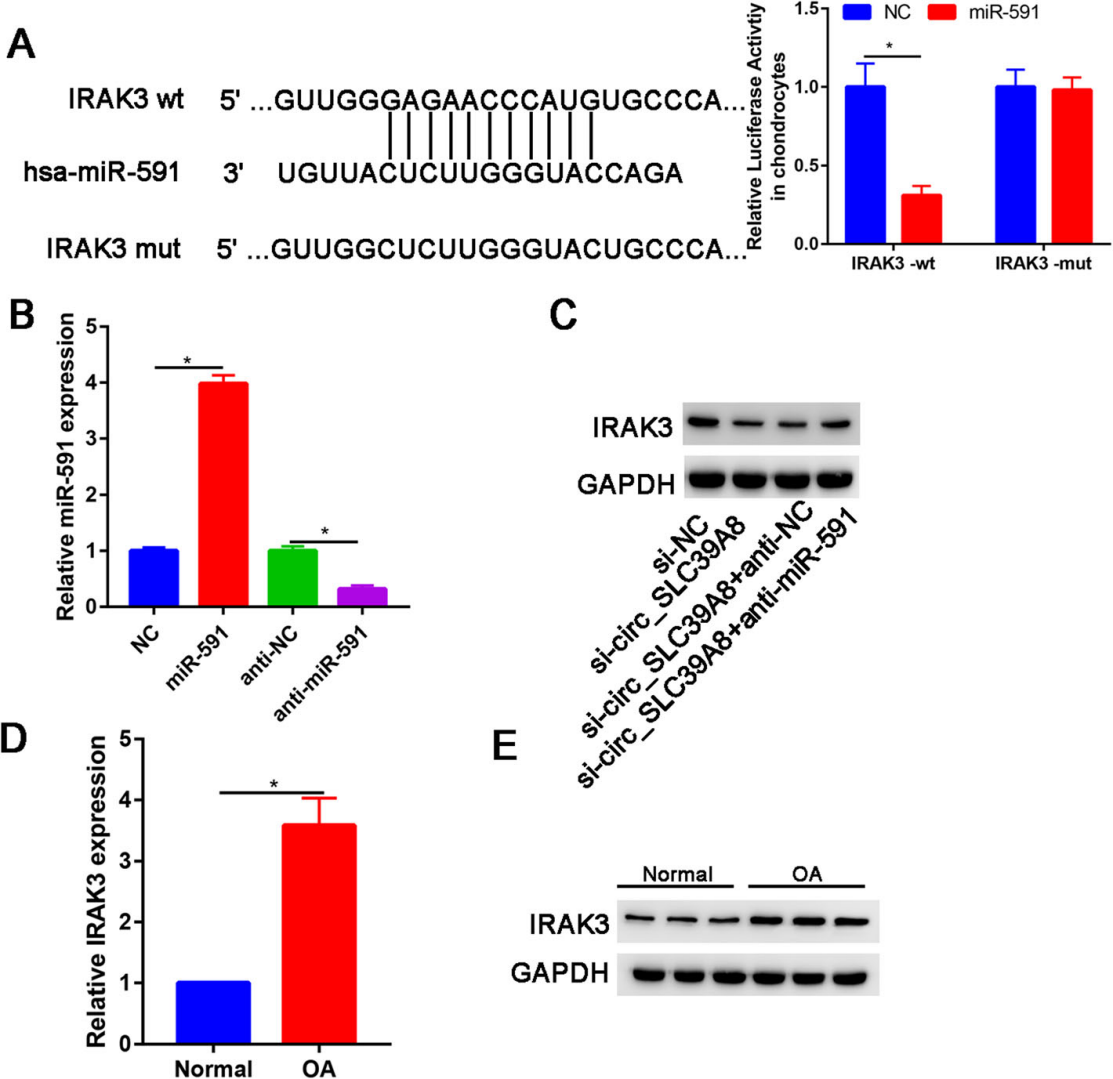
IRAK3 was a downstream target of miR-591. **a** The predicted binding sites of miR-591 in the 3′UTR of IRAK3 mRNA were identified by using the starBase database and the relative luciferase activity in chondrocytes co-transfected with wt-IRAK3 or mut-IRAK3 and miR-591 mimic or miRNA NC was determined by dual-luciferase reporter assay. **b** The level of miR-591 was determined in OA chondrocytes transfected with NC, miR-591, anti-NC, or anti-miR-591. **c** The level of IRAK3 protein expression level was determined in OA chondrocytes transfected with NC, miR-591, anti-NC, or anti-miR-591. **d** The IRAK3 mRNA expression in OA cartilage and normal cartilage. **e** The IRAK3 protein expression in OA cartilage and normal cartilage. *P<0.05

The results indicated that the luciferase activity of IRAK3-wt was markedly reduced post-transfection with miR-591; however, no change was observed in the activity of cells transfected with IRAK3-mut in the presence of miR-591 (Fig. 5a).

The results of qRT-PCR showed that the expression of miR-591 was increased and decreased in OA chondrocytes transfected with miR-591 and anti-miR-591, respectively (Fig. 5b), indicating that transfection of miR-591 and anti-miR-591 was successful.

Next, western blot was performed to assess the IRAK expression after knockdown of circ_SLC39A8 and knockdown of miR-591 (Fig. 5c). We found that the IRAK3 expression was significantly downregulated after silencing circ_SLC39A8 and significantly increased after silencing miR-591. IRAK3 mRNA (Fig. 5d) and protein expression (Fig. 5e) were assessed by RT-PCR and western blot, respectively. We found that IRAK3 expression was significantly upregulated in OA cartilage than normal cartilage.

### Overexpression IRAK3 partially reversed the miR-591 on chondrocyte apoptosis, matrix degradation, and inflammation response

**Fig. 6:**
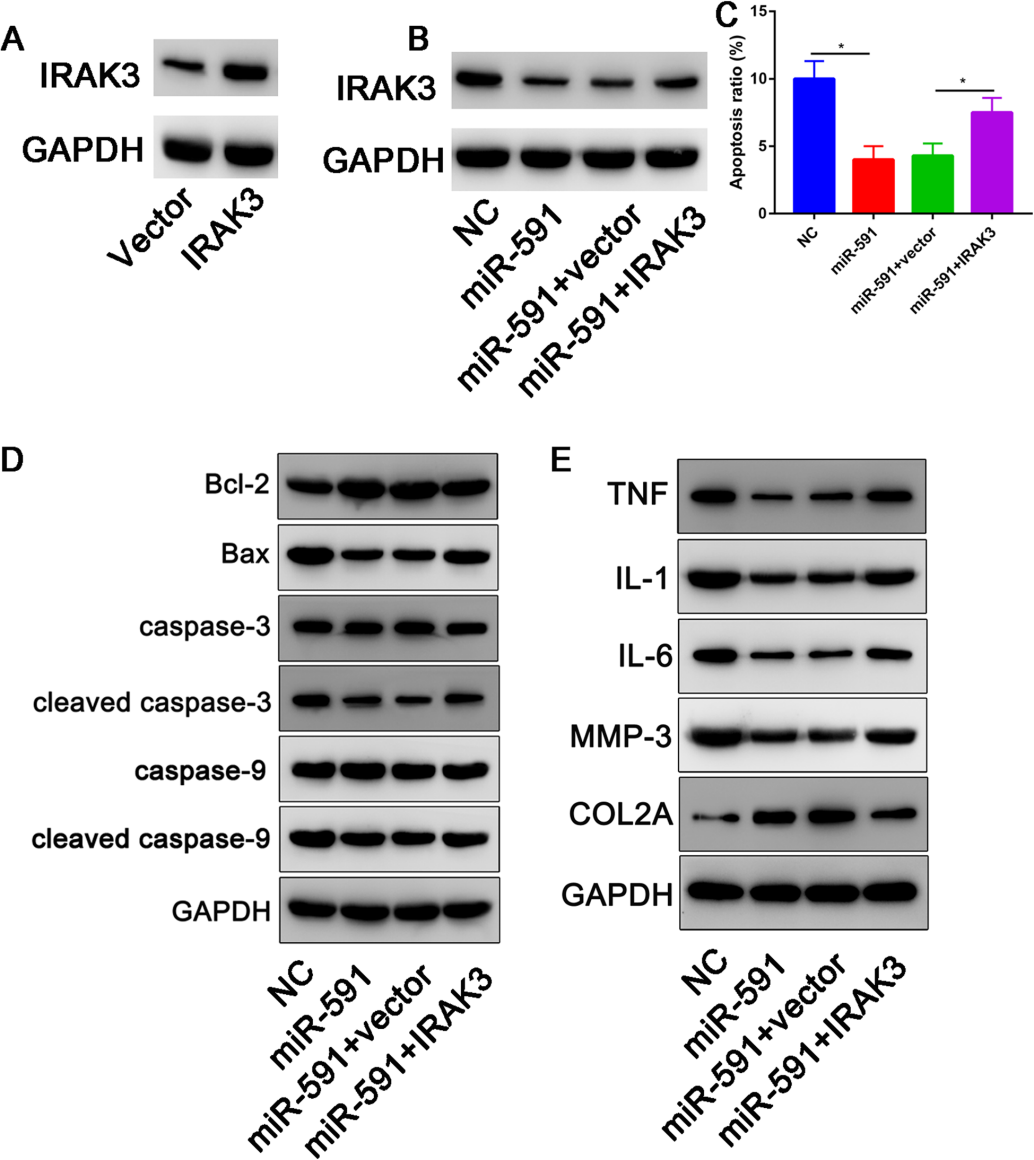
Overexpression of IRAK3 partially reversed miR-591 on chondrocyte apoptosis. **a** Overexpression efficiency of IRAK3 was determined by western blot assay in OA chondrocytes transfected with IRAK3 or vector. **b** IRAK expression in OA chondrocytes transfected with NC, miR-591, miR-591 + vector, or miR-591 + IRAK3. **c** Cell apoptosis was determined using flow cytometry analysis. **d** The protein levels of Bcl-2, Bax, caspase 3, cleaved-caspase 3, caspase 9, and cleaved-caspase 9 were analyzed by western blot assay. **e** Western blot assay was carried out to examine the protein expression of TNF-α, IL-1β, IL-6, MMP3, and COL2A1. *P<0.05

Western blot assay was used to detect the transfection efficiency of IRAK3. The data showed that IRAK3 was successfully overexpressed after transfection with IRAK3 (Fig. 6a). To explore whether miR-591 exerted its biological functions by targeting IRAK3, OA chondrocytes were transfected with NC, miR-591, miR-591 + vector, or miR-591 + IRAK3. Overexpression of miR-591 inhibited the protein expression of IRAK3, which was restored by the addition of IRAK3 (Fig. 6b). miR-591 restoration increased the protein level of Bcl-2 and decreased the protein expression of Bax expression, C-caspase 3/caspase 3 ratio, and c-caspase 9/caspase 9 ratio, whereas these effects were abated by upregulating IRAK3 (Fig. 6c). Furthermore, the protein levels of TNF-α, IL-1β, IL-6, and MMP3 were reduced, and collagen type II expression was increased after transfection with miR-591, while co-transfection with IRAK3 abolished these effects (Fig. 6d).

## Discussion

In the present study, we first identified circ_SLC39A8 as a key upregulated circRNA involved in OA. In addition, we used gain-of-function and loss-of-function approaches in vitro to demonstrate the participation of circ_SLC39A8 in the proapoptotic response and protection of ECM components from degradation. We revealed a new function of circ_SLC39A8 in OA progression and identified it as a promising therapeutic target for OA treatment.

Recent evidence indicates that circular RNA is a special type of non-coding RNAs that is widely expressed in human cells and participates in the regulation of transcription and post-transcriptional genes [27]. In recent years, more and more evidences have shown that circRNAs have different biological functions and new RNA molecules of pathological significance [11]. The most reported function is that circRNAs act as miRNA sponges to regulate miRNA and the expression of its downstream target genes. For example, Zhou et al. [28] found that circRNA.33186 contributes to the pathogenesis of OA by sponging miR-127-5p. Huang et al. [29] revealed that circRNA_0092516 regulates chondrocyte proliferation and apoptosis in OA through the miR-337-3p/PTEN axis. Chen et al. [30] revealed that circRNA-UBE2G1 regulates LPS-induced OA through the miR-373/HIF-1a axis. However, these studies were insufficiently reliable to add significantly to the existing literature. In this study, we found that circ_SLC39A8 was significantly upregulated in OA cartilage tissue, which was validated by qRT-PCR. Circ_SLC39A8 is generated by the back splicing of the exons 4 of the SLC39A8 gene. We used several gain/loss of function experiments to confirm the mechanism of circ_SLC39A8 for regulating chondrocytes apoptosis in vitro. Since IL-1β is an important factor in the regulation of inflammatory response in the pathogenesis of OA, we used IL-1β to construct in vitro research model of OA. We found that circ_SLC39A8 was significantly upregulated in OA patients and treatment with RNaseR enriches for circular RNAs in contrast to linear RNAs. Because circular RNAs are RNaseR resistant, treatment with RNaseR enriches for circular RNAs in contrast to linear RNAs. According to reports, circular RNA can act as a microRNA sponge.

miRNAs, a series of endogenous small non-coding RNAs with approximately 18–22 nucleotides in length, have been reported to be involved in diverse diseases, including OA [31–34]. We found that circ_SLC39A8 mainly through targeting with miR-591, which validated through bioinformatic analysis and luciferase reporter assay. What is more, knockdown of miR-591 could partially block the effects of si-circ_SLC39A8 on chondrocyte apoptosis. All these findings suggested that circ_SLC39A8 targeting miR-591 to regulate the chondrocytes apoptosis. Previous study found that miR-591 could regulate hepatocellular carcinoma apoptosis through regulating FOSL2 expression [21]. And the miR-591/HK2 axis could also regulate breast cancer apoptosis [23]. Previous studies suggest that circRNAs target the miRNA as a miRNA sponge and bind to its target and modulate the cellular function [35].

In this study, we further explored the target of miR-591. Through bioinformatic analysis, we found that miR-591 has putative binding sites with IRAK3, which was validated by luciferase reporter assay. A small number of studies showed that tumor cell-intrinsic IRAK3 could also support the progression of tumor cells in colorectal and lung cancers [24]. In this study, we found that overexpression of IRAK3 could partially reverse the effects of miR-591 overexpression on chondrocyte apoptosis.

However, the limitations of the present study are that the findings were not confirmed in vivo in animal models. One miRNA may regulate many genes as its targets, while one gene may be targeted by many miRNAs. Thus, the target gene of miR-591 needs for more studies to verify. Since the molecular mechanisms involved in the pathogenesis of OA have not yet been fully clarified, current treatment methods can only relieve symptoms without preventing further cartilage destruction. Therefore, an in-depth study of its pathogenesis is essential to clarify the occurrence and development of OA.

## Conclusion

In conclusion, our study revealed that knockdown of circ_SLC39A8 could inhibit the chondrocyte apoptosis, matrix degradation, and inflammation response. The potential mechanism of knockdown of circ_SLC39A8 for chondrocyte apoptosis was that through mediating the miR-591/IRAK3 axis. Our study is the first study to comprehensively understand the role of circ_SLC39A8/miR-591/IRAK3 in OA chondrocytes.

## Data Availability

We state that the data will not be shared since all the raw data are present in the figures included in the article.

## Abbreviations

circRNAs: Circular RNAs
OA: Osteoarthritis
qRT-PCR: Quantitative real-time polymerase chain reaction
CCK-8: Cell Counting Kit-8
IRAK3: Interleukin-1-receptor-associated kinase 3
ECM: Extracellular matrix
OARSI: Osteoarthritis Research Society International
ceRNAs: Competitive endogenous RNAs
miRNA: microRNA
IRAK: IL receptor-associated kinase
siRNAs: Small interfering RNAs
miR-591: miR-591 mimic
anti-miR-591: miR-591 inhibitor
FITC: Annexin V-fluorescein isothiocyanate
PI: Propidium iodide
RIPA: Radioimmunoprecipitation
PMSF: Phenylmethylsulfonyl fluoride
SDS-PAGE: Sodium dodecyl sulphate-polyacrylamide gel electrophoresis
PVDF: Polyvinylidene fluoride
TBST: Tris-buffered saline Tween-20
ECL: Enhanced chemiluminescence
wt: Wild-type
mut: Mutant

## References

[1] Zheng L, Zhang Z, Sheng P, et al. The role of metabolism in chondrocyte dysfunction and the progression of osteoarthritis. Ageing Res Rev. 2020;101249. 10.1016/j.arr.2020.101249. 10.1016/j.arr.2020.101249 33383189

[2] Nelson AE (2018). Osteoarthritis year in review 2017: clinical. Osteoarthritis Cartilage.

[3] Flemming DJ, Gustas-French CN (2017). Rapidly progressive osteoarthritis: a review of the clinical and radiologic presentation. Curr Rheumatol Rep.

[4] Xia B, Di C, Zhang J (2014). Osteoarthritis pathogenesis: a review of molecular mechanisms. Calcif Tissue Int.

[5] Watt FE (2018). Osteoarthritis biomarkers: year in review. Osteoarthritis Cartilage.

[6] Geyer M, Schönfeld C (2018). Novel insights into the pathogenesis of osteoarthritis. Curr Rheumatol Rev.

[7] Palazzo C, Nguyen C, Lefevre-Colau MM (2016). Risk factors and burden of osteoarthritis. Ann Phys Rehabil Med.

[8] Scanzello CR (2017). Role of low-grade inflammation in osteoarthritis. Curr Opin Rheumatol.

[9] Greene MA, Loeser RF (2015). Aging-related inflammation in osteoarthritis. Osteoarthritis Cartilage.

[10] Du WW, Zhang C, Yang W (2017). Identifying and characterizing circRNA-protein interaction. Theranostics.

[11] Chen LL, Yang L (2015). Regulation of circRNA biogenesis. RNA Biol.

[12] Jiang S, Liu Y (2020). Noncoding RNAs: new regulatory code in chondrocyte apoptosis and autophagy. Wiley Interdiscip Rev RNA.

[13] Jiang R, Gao H, Cong F (2020). Circ_DHRS3 positively regulates GREM1 expression by competitively targeting miR-183-5p to modulate IL-1β-administered chondrocyte proliferation, apoptosis and ECM degradation. Int Immunopharmacol.

[14] Fu Q, Li L, Wang B, et al. CircADAMTS6/miR-431-5p axis regulate interleukin-1β induced chondrocyte apoptosis. J Gene Med. 2020:e3304. 10.1002/jgm.3304. 10.1002/jgm.3304 33305412

[15] Zhou JL, Deng S, Fang HS, et al. Circular RNA circANKRD36 regulates Casz1 by targeting miR-599 to prevent osteoarthritis chondrocyte apoptosis and inflammation. J Cell Mol Med. 2020. 10.1111/jcmm.15884. 10.1111/jcmm.15884 PMC7810932 33205602

[16] Wu Y, Lu X, Shen B (2019). The therapeutic potential and role of miRNA, lncRNA, and circRNA in osteoarthritis. Curr Gene Ther.

[17] Zhong G, Long H, Ma S (2019). miRNA-335-5p relieves chondrocyte inflammation by activating autophagy in osteoarthritis. Life Sci.

[18] Coutinho de Almeida R, Ramos YFM, Mahfouz A (2019). RNA sequencing data integration reveals an miRNA interactome of osteoarthritis cartilage. Ann Rheum Dis.

[19] Gargano G, Oliviero A, Oliva F, et al. Small interfering RNAs in tendon homeostasis. Br Med Bull. 2021. 10.1093/bmb/ldaa040. 10.1093/bmb/ldaa040 33454750

[20] Huang X, Tang F, Weng Z (2019). MiR-591 functions as tumor suppressor in breast cancer by targeting TCF4 and inhibits Hippo-YAP/TAZ signaling pathway. Cancer Cell Int.

[21] Ji C, Hong X, Lan B, et al. Circ_0091581 promotes the progression of hepatocellular carcinoma through targeting miR-591/FOSL2 axis. Dig Dis Sci. 2020. 10.1007/s10620-020-06641-4. 10.1007/s10620-020-06641-4 33040214

[22] Sucharov CC, Kao DP, Port JD (2017). Myocardial microRNAs associated with reverse remodeling in human heart failure. JCI Insight.

[23] Xing Z, Wang X, Liu J, et al. Hsa_circ_0069094 accelerates cell malignancy and glycolysis through regulating the miR-591/HK2 axis in breast cancer. Cell Signal. 2020;109878. 10.1016/j.cellsig.2020.109878. 10.1016/j.cellsig.2020.109878 33309838

[24] Wu X, Ouyang Y, Wang B (2020). Hypermethylation of the IRAK3-activated MAPK signaling pathway to promote the development of glioma. Cancer Manag Res.

[25] Liu Y, Lin L, Zou R (2018). MSC-derived exosomes promote proliferation and inhibit apoptosis of chondrocytes via lncRNA-KLF3-AS1/miR-206/GIT1 axis in osteoarthritis. Cell Cycle.

[26] Zhang B, Sun M, Wang J (2019). MiR-671 ameliorates the progression of osteoarthritis in vitro and in vivo. Pathol Res Pract.

[27] Zhang HD, Jiang LH, Sun DW (2018). CircRNA: a novel type of biomarker for cancer. Breast Cancer.

[28] Zhou ZB, Huang GX, Fu Q (2019). circRNA.33186 Contributes to the pathogenesis of osteoarthritis by sponging miR-127-5p. Mol Ther.

[29] Huang Z, Ma W, Xiao J, et al. CircRNA_0092516 regulates chondrocyte proliferation and apoptosis in osteoarthritis through the miR-337-3p/PTEN axis. J Biochem. 2020. 10.1093/jb/mvaa119. 10.1093/jb/mvaa119 33135071

[30] Chen G, Liu T, Yu B (2020). CircRNA-UBE2G1 regulates LPS-induced osteoarthritis through miR-373/HIF-1a axis. Cell Cycle.

[31] Chen L, Li Q, Wang J (2017). MiR-29b-3p promotes chondrocyte apoptosis and facilitates the occurrence and development of osteoarthritis by targeting PGRN. J Cell Mol Med.

[32] Wang Z, Hu J, Pan Y (2018). miR-140-5p/miR-149 affects chondrocyte proliferation, apoptosis, and autophagy by targeting FUT1 in osteoarthritis. Inflammation.

[33] Giordano L, Porta GD, Peretti GM (2020). Therapeutic potential of microRNA in tendon injuries. Br Med Bull.

[34] Oliviero A, Della Porta G, Peretti GM (2019). MicroRNA in osteoarthritis: physiopathology, diagnosis and therapeutic challenge. Br Med Bull.

[35] Salzman J (2016). Circular RNA expression: its potential regulation and function. Trends Genet.

